# The Impact of COVID-19 on Risky Behaviors and Health Changes in African-American Smokers Who Are Eligible for LDCT Screening

**DOI:** 10.3389/fpubh.2021.745925

**Published:** 2021-12-08

**Authors:** Tung-Sung Tseng, Mirandy Li, Yu-Hsiang Kao, Lei-Shih Chen, Hui-Yi Lin

**Affiliations:** ^1^Behavioral and Community Health Sciences Program, School of Public Health, Louisiana State University Health Sciences Center, New Orleans, LA, United States; ^2^Department of Health and Kinesiology, Texas A&M University, College Station, TX, United States; ^3^Biostatistics Program, School of Public Health, Louisiana State University Health Sciences Center, New Orleans, LA, United States

**Keywords:** smoking, risky behavior, LDCT lung cancer screening, COVID-19, African American (AA), healthy behavior

## Abstract

The COVID-19 pandemic has disrupted much of day-to-day life in the US and around the world. Smokers have a higher risk of adverse outcomes due to COVID-19. This study investigated the impact of COVID-19 on risky behaviors and health changes in lower income African-American smokers eligible for Low dose computed tomography (LDCT) screening, who may be more adversely impacted by the COVID-19 pandemic. A total of 22 African-American daily smokers who were eligible for LDCT screening participated in this study. The mean age of participants was 61.2 years old (SD = 4.7), 77.3% of the smokers were female, all participants had an income below $20,000, and 63.6% were on Medicaid. Descriptive statistics were used to provide summary information on demographics, COVID-19, and health status. Results showed that participants increased cigarette smoking, spent more time on screens, increased sugary drink consumption, consumed more vegetables and fruits, and engaged in more gardening activities during the COVID-19 pandemic. However, participants also decreased physical activity time and slept less during the pandemic. In general, more than one-third of participants gained more body weight and reported increased stress and anxiety. Our results suggest that African-American smokers who qualify for LDCT screening should be encouraged to consider strategies not only for smoking cessation, but also risky behavior control and management.

## Introduction

The COVID-19 pandemic has disrupted much of day-to-day life in the US and around the world (1). The first case of COVID-19 was identified in the US on January 20, 2020, and has resulted in ~32 million cases and 600,000 deaths in the US, as of May 2021 (2). Smoking has been associated with a greater risk of adverse outcomes in COVID-19, with current and former smokers being 1.4 times more likely to suffer severe symptoms of COVID 19, and 2.4 times more likely to need ICU support, mechanical ventilation, or die, compared to non-smokers (3). However, some studies have shown that smokers tend to increase the frequency of smoking behaviors during quarantine, which further increases the negative health risks associated with smoking (4). In a study on a predominantly African-American population of patients with COVID-19, those who were smokers showed an increased need of ICU admission and higher mortality (5). Furthermore, African-Americans are more likely to have comorbid conditions such as hypertension, diabetes, and obesity, all of which may exacerbate COVID-19 outcomes (6). During the COVID-19 pandemic, African-Americans in a US national survey were more likely than White Americans to leave their homes, possibly due to social circumstances or lower access to accurate knowledge of how the disease is spread (7).

African-Americans and White Americans smoke at comparable rates; however, when compared to White smokers, African-American smokers smoke fewer cigarettes per day, and initiate smoking at a later age (8). Despite this, African-American smokers bear a disproportionate burden of smoking-related diseases (8). African-American smokers are also more likely than White smokers to want to quit and attempt to quit, but are less likely to quit successfully than White smokers (9). This may be due to lower access and utilization of smoking cessation services (9). Despite knowledge about the increased risk of COVID-19 on smokers and the health disparities facing African-American smokers, smoking behaviors during the COVID-19 pandemic have not been well-investigated in this population. Currently, the smoking behaviors of African-American smokers during COVID-19 are unclear.

In addition to smoking, changes in other health behaviors due to COVID-19 are also of concern. Previous studies have demonstrated increased weight gain, decreased physical activity, decreased consumption of fruit and vegetables, increased screen time, decreased sleep, and increased alcohol consumption during quarantine (4, 10–13). Risky behaviors, such as smoking, are likely to cluster with other risky behaviors, such as alcohol use (14). In a separate nationally representative survey of 604 African-Americans, fewer than 80% of participants reported adhering to the four COVID-19 public health recommendations of frequently washing hands for at least 20 s, staying 6 or more feet apart from others in public, avoiding touching the face, and wearing a mask when in contact with others (15). In Louisiana, African-Americans represent 32.2% of the state population, but 70.5% of COVID-19 deaths are African-American (16). This disparity may be due to several risk factors facing the African-American population in Louisiana, such as higher prevalence of chronic disease, lower access to knowledge about COVID-19, increased shelter insecurity, and other factors (7). Because of this stark disparity, it is important to understand the behavioral changes of African-American smokers during COVID-19. The objective of this study was to investigate the impact of COVID-19 on risky behaviors and health changes in lower income African-American smokers eligible for Low dose computed tomography (LDCT) screening, who may be more adversely impacted by the COVID-19 pandemic.

## Methods

### Study Design and Population

This study applied a cross-sectional design via phone survey in 2020. Eligible participants were African Americans smokers ages 55–77 years old have who had either had 30+ pack-years of smoking or had received an LDCT exam in the past year, who were eligible for LDCT screening in primary care clinics at a large hospital in New Orleans, LA. A total of 22 African-American daily smokers agreed to participate in the survey via phone. Enrollees were asked to give verbal consent to confirm that they understood the rights and privacy protection disclaimer before the survey. After completing the survey, an e-informed consent document was distributed to all participants for their records. The survey was an anonymous questionnaire that collected demographic information, COVID-19 and health status, and behavior related information. This study protocol was reviewed and approved by the Institutional Review Board of LSUHSC-NO (approval #10104).

### Subject Demographics

Participant demographics included age, gender, income, education, marriage status, and insurance type. Gender was classified as female or male. Income level included two categories (< $20,000, and equal to, or higher than $20,000). Education level was classified into four groups (Grades 1 through 8, Grades 9 through 11, Grade 12 or GED, and College 1–3 years). Marital status was classified into four categories [married, divorced, widowed, and single (i.e., never married and not now living with a partner)]. Insurance type included health insurance (private health insurance, Blue Cross and Blue Shield, and HMOs), Medicaid, Medicare, paid with cash, and others.

### COVID-19 and Health Status

There were three questions pertaining to COVID-19, including “Have you been diagnosed positive for COVID-19 in the recent months?”(Yes/No), “If Yes, did you stay at a hospital to treat COVID-19?”(Yes/No), and “How likely do you think it is that you will get COVID-19 in the future?” (very low to very high, using a 5-point Likert scale). Health status included body mass index (BMI) and chronic disease history. BMI was classified into normal weight (<25 kg/m^2^), overweight (25–29.9 kg/m^2^), and obese (≥30 kg/m^2^). Participants were asked the question “Have you been diagnosed with any of the following chronic diseases?” to identify their chronic disease history [responses included hypertension, diabetes, asthma, chronic obstructive pulmonary disease (COPD), mental health disorder, coronary heart disease, arthritis, kidney disease, liver disease, and cancer].

### Behavioral Changes During the COVID-19 Pandemic

We assessed behavioral changes via the question “Did you change the following behaviors during the COVID-19 pandemic?” Responses included twelve behaviors: cigarette smoking, alcohol consumption, vegetable and fruit consumption, sugary drink consumption, exercise times, sleep hours, screen time, vitamin intake, gardening, body weight, stress, and anxiety. Each question had five levels of response (decrease, slightly decrease, no change, slight increase, and increase).

### Statistical Analyses

Descriptive statistics were used to provide summary information on demographics, COVID-19, and health status. All analyses were performed using SAS version 9.4 (SAS Institute, Cary, NC, USA).

## Results

The demographic characteristics of all participants are shown in Table 1. The mean age of participants was 61.2 years old (SD = 4.7). As shown in Table 1, 77.3% of participants were female, and all participants reported an income below $20,000. Half of participants reported an education of high school or below, half reported being single (i.e., never married and not now living with a partner), and 63.6% were on Medicaid.

**Table 1:** Participant demographics (*n* = 22).

**Variables**	***n* (%)**
Age, Mean (SD)	61.2 (4.7)
**Gender**	
Female	17 (77.3)
Male	5 (22.7)
**Annual income**	
<$20,000	22 (100.0)
**Education level**	
Grades 1 through 8	1 (4.6)
Grades 9 through 11	10 (45.5)
Grade 12 or GED	8 (36.4)
College 1–3 years	3 (13.6)
**Marital status**	
Married	2 (9.1)
Divorced	7 (31.8)
Widowed	2 (9.1)
Single^#^	11 (50.0)
**Insurance**	
Paid with cash	4 (18.2)
Health insurance^$^	2 (9.1)
Medicaid	14 (63.6)
Medicare	8 (36.4)
Others	1 (4.6)

# *Never married and not now living with a partner*.

$ *Private health insurance, Blue Cross and Blue Shield, HMOs*.

Table 2 shows COVID-19 and health status. Two smokers (10%) had been diagnosed positive for COVID-19, and one of them was treated in a hospital. Eighteen smokers (90%) felt that they had a very low or low risk of getting COVID-19 in the future. In terms of health status, 72.7% of participants were overweight or obese. More than half of smokers mentioned that they had been diagnosed with hypertension, diabetes, or arthritis. About one-third had asthma or COPD.

**Table 2:** COVID-19 and Health Status (*n* = 22).

**Variables**	***n* (%)**
**COVID-19**	
Have you been diagnosed positive for COVID-19 in the recent months?	
No	19 (90.5)
Yes	2 (9.5)
If YES, did you stay at a hospital to treat COVID-19?	
No	1 (50.0)
Yes	1 (50.0)
How likely do you think it is that you will get COVID-19 in the future?	
Very low	13 (65.0)
Somewhat low	5 (25.0)
Moderate	1 (5.0)
Somewhat high	0 (0.0)
Very high	1 (5.0)
**Health Status**	
Body Mass Index	
Normal (18.5–24.9 kg/m^2^)	6 (27.3)
Overweight (25–29.9 kg/m^2^)	7 (31.8)
Obese (≧30 kg/m^2^)	9 (40.9)
Have you been diagnosed with the following chronic diseases?	
Hypertension	10 (47.6)
Diabetes	11 (52.4)
Asthma	5 (23.8)
COPD	6 (28.6)
Mental health disorder	2 (9.5)
Coronary heart disease	4 (19.1)
Arthritis	12 (57.1)
Kidney diseases	1 (4.8)
Liver disease	3 (14.3)
Cancer	1 (4.8)

Behavioral changes during the COVID-19 pandemic are shown in Table 3. Regarding smoking behavior, 42.9% of participants reported increased cigarette smoking and 28.6% of participants reported decreased cigarette smoking during the COVID-19 pandemic. For alcohol intake, only 19% of participants reported that they consumed more alcohol during the pandemic. Additionally, 38.1% of participants consumed more vegetables and fruits, and around 40% of participants reported that they engaged in more gardening activities. However, 33.3% of participants increased sugary drink intake, 23.8% of participants decreased physical activity time, and 42.9% of participants spent more time on screens (including TVs, smartphones, tablets, and computers). Over 30% of participants gained more body weight, slept less, and felt increased stress and anxiety during the pandemic.

**Table 3:** Behavior and health changes (*n* = 22).

**Variable**	***n* (%)**
**DID YOU CHANGE THE FOLLOWING BEHAVIORS DURING THE COVID-19 PANDEMIC?**	
**Cigarette smoking**	
Decrease	2 (9.5)
Slightly decrease	4 (19.1)
No change	6 (28.6)
Slightly increase	5 (23.8)
Increase	4 (19.1)
**Alcohol consumption**	
Decrease	0 (0.0)
Slightly decrease	1 (4.8)
No change	15 (71.4)
Slightly increase	2 (9.5)
Increase	2 (9.5)
NA	1
**Vegetable and fruit consumption**	
Decrease	2 (9.5)
Slightly decrease	1 (4.8)
No change	10 (47.6)
Slightly increase	3 (14.3)
Increase	5 (23.8)
**Sugary drink consumption**	
Decrease	1 (4.8)
Slightly decrease	0 (0.0)
No change	13 (61.9)
Slightly increase	5 (23.8)
Increase	2 (9.5)
**Exercise time**	
Decrease	3 (14.3)
Slightly decrease	2 (9.5)
No change	13 (61.9)
Slightly increase	2 (9.5)
Increase	1 (4.8)
**Sleep hours**	
Decrease	2 (9.5)
Slightly decrease	4 (19.1)
No change	11 (52.4)
Slightly increase	2 (9.5)
Increase	2 (9.5)
**Screen time (i.e., TV, phone, PC, tablet)**	
Decrease	1 (4.8)
Slightly decrease	0 (0.0)
No change	11 (52.4)
Slightly increase	4 (19.1)
Increase	5 (23.8)
**Vitamin intake**	
Decrease	3 (14.3)
Slightly decrease	0 (0.0)
No change	16 (76.2)
Slightly increase	1 (4.8)
Increase	0 (0.0)
NA	1 (4.8)
**Gardening**	
Decrease	0 (0.0)
Slightly decrease	1 (4.8)
No change	12 (57.1)
Slightly increase	4 (19.1)
Increase	4 (19.1)
**Body weight**	
Decrease	0 (0.0)
Slightly decrease	1 (4.8)
No change	12 (57.1)
Slightly increase	4 (19.1)
Increase	4 (19.1)
**Stress**	
Decrease	1 (4.8)
Slightly decrease	0 (0.0)
No change	11 (52.4)
Slightly increase	5 (23.8)
Increase	4 (19.1)
**Anxiety**	
Decrease	1 (4.8)
Slightly decrease	1 (4.8)
No change	12 (57.1)
Slightly increase	3 (14.3)
Increase	4 (19.1)

## Discussion

Most African-American smokers eligible for LDCT screening reported increased cigarette smoking during the COVID-19 pandemic, although about one-third reported decreasing cigarette smoking. This finding is consistent with some previous studies that also reported decreases in cigarette consumption during COVID-19 quarantine periods (17, 18). One previous study reported that smoking was associated with a greater risk of adverse outcomes in COVID-19, with current and former smokers being 2.4 times as likely to need ICU support or die, compared to non-smokers (3). Although this study focused on smoking and other risky behavioral changes during the pandemic, it should be noted that African-American smokers may be more adversely impacted by the COVID-19 pandemic. A recent study showed that African-Americans are more likely to have comorbid conditions such as hypertension, diabetes, and obesity, all of which may exacerbate COVID-19 outcomes (6). Our results also showed that 72.7% of African-American smokers in this study were overweight or obese. Furthermore, more than half of participants mentioned that they have been diagnosed with hypertension, diabetes, or arthritis, which may lead to more adverse COVID-19 related outcomes.

Our results also showed that 38% of participants experienced an increase in bodyweight during the COVID-19 pandemic. This may have resulted from increased sugary drink intake, increased screen time, and decreased physical activity. Compared to pre-pandemic, screen time increased (42%) and exercise time decreased (23.8%) during the pandemic. Previous studies have shown that individuals reported higher levels of screen time during the pandemic, which was associated with poorer mental health (19). Additionally, another study demonstrated that COVID-19 home lockdown was associated with a decrease in physical activity levels, as well as unhealthier eating patterns (20). However, African-American smokers in this study consumed more vegetables and fruits during the pandemic, and around 40% of participants reported they engaged in more gardening activities. Home gardening has been shown to increase food security and the intake of nutritious foods, and has been suggested as a potential strategy to combat food insecurity in areas affected by COVID-19 labor shortages (21). These results suggest that the COVID-19 pandemic can influence smokers' eating, home gardening and food consumption behaviors.

Another important finding in this study is that participants reported sleeping less and feeling more stress and anxiety during the pandemic. These mental health changes could be due to the perception of potential illness and life challenges exacerbated by the COVID-19 pandemic. A recent systematic review demonstrated that the COVID-19 pandemic has resulted in significant negative psychological impacts, and contributed to a concurrent mental health epidemic (22). Consistent with our findings, several studies have also shown changes in sleep, stress, and anxiety as a result of the pandemic (23–25).

It is important to note that there are some limitations inherent to this study. First, this is a pilot study using self-reported data, which tends to give narrow estimated associations. Second, the small sample size from a single hospital targeting lower income African-American smokers limits the generalizability and reliability of results. Third, the behavioral changes were only collected at one time point during the pandemic, and may have changed as the pandemic progressed. Despite these limitations, this study focuses on lower income, higher risk African-American smokers who may be more adversely impacted by the COVID-19 pandemic during the time of data collection.

## Conclusion

This study found that African-American smokers who were eligible for LDCT screening increased cigarette smoking, spent more time on screens, increased sugary drink consumption, consumed more vegetables and fruits, and engaged in more gardening activities during the COVID-19 pandemic. However, participants also decreased time spent pursuing physical activities and slept less. In general, more than one-third of participants gained more body weight and felt increased stress and anxiety. Our results suggest that African-American smokers who qualify for LDCT screening should be encouraged to consider strategies not only for smoking cessation, but also risky behavior control and management.

## Data Availability Statement

The original contributions presented in the study are included in the article and supplementary material. Further inquiries can be directed to the corresponding author(s).

## Ethics Statement

The studies involving human participants were reviewed and approved by the Institutional Review Board of LSUHSC-NO (approval #10104). The patients/participants provided their written informed consent to participate in this study.

## Author Contributions

T-ST contributed to the study conception and drafted the first manuscript. ML reviewed the manuscript and conducted the literature review. H-YL checked quality and results. T-ST, Y-HK, and L-SC contributed to the data analysis and the interpretation of the study. All authors contributed to editing and revising the manuscript critically and approved the final version of the article to be published.

## Funding

This study was supported by The Louisiana Cancer Research Consortium, Louisiana Tobacco Control Initiative and LSUHSC School of Public Health.

## Conflict of Interest

The authors declare that the research was conducted in the absence of any commercial or financial relationships that could be construed as a potential conflict of interest.

## Publisher's Note

All claims expressed in this article are solely those of the authors and do not necessarily represent those of their affiliated organizations, or those of the publisher, the editors and the reviewers. Any product that may be evaluated in this article, or claim that may be made by its manufacturer, is not guaranteed or endorsed by the publisher.
